# NON-ARTERITIC ANTERIOR ISCHEMIC OPTIC NEUROPATHY SECONDARY TO TADALAFIL USE

**DOI:** 10.51894/001c.122862

**Published:** 2024-08-30

**Authors:** Paige Mellema, Blake Deutmeyer, Paul Wasielewski

**Affiliations:** 1 Neurology & Medicine University of Michigan Health West

## 22

### INTRODUCTION

Erectile dysfunction (ED) affects over 150 million men globally, especially prevalent in older age groups. The primary treatment involves FDA-approved phosphodiesterase-5 inhibitors (PDE5Is) like sildenafil, tadalafil, vardenafil, and avanafil. These drugs enhance smooth muscle relaxation in the corpus cavernosum, facilitating penile erection by increasing Guanosine 3’,5’-cyclic monophosphate levels. Despite effectiveness, PDE5Is have non-selective actions, potentially causing systemic side effects. Non-arteritic anterior ischemic optic neuropathy (NAION), a primary cause of optic nerve swelling in those aged 50 and older, may result from inadequate blood supply to the optic nerve. Medications like PDE5 inhibitors, enhancing nitric oxide impact, may contribute to nocturnal hypotension and insufficient blood flow to the posterior ciliary arteries, increasing NAION risk.

### CASE DESCRIPTION

A 62-year-old male with bilateral papilledema was referred to the emergency department after routine optometry revealed optic disc abnormalities. The patient’s medical history included gout, hyperlipidemia, diabetes, hypertension, and coronary artery disease, with current medications like allopurinol, aspirin, atorvastatin, lisinopril, metformin, and semaglutide. Despite a thorough examination, no acute issues were found. Neuroimaging studies, including MRI brain and venogram, showed no acute pathological findings, with MRI revealing no signs of infarct, hemorrhage, fluid collection, mass, shift, or hydrocephalus. Lumbar puncture indicated elevated CSF protein (114 mg/dL) and glucose (90 mg/dL), but negative serological tests and normal serum protein electrophoresis ruled out malignancies and acute abnormalities in chest, abdomen, and pelvis CT. Increased Cialis use during a recent vacation coincided with vision loss, leading to the diagnosis of NAION secondary to PDE5I use. The patient was discharged with a neuro-ophthalmologist referral, emphasizing the importance of considering medication-related causes in optic neuropathy cases.

### DISCUSSION/CONCLUSION

Existing literature recognizes the potential association between PDE5Is and NAION, with risk factors including hypertension, hypercholesterolemia, diabetes, and cardiovascular diseases. The patient’s history aligned with documented associations, emphasizing cautious PDE5I use. Studies like the Ischemic Optic Neuropathy Decompression Trial provide insights into surgical interventions but lack conclusive evidence. Clinicians must be vigilant about medication-induced complications, educating patients on possible ocular side effects. Further research is essential to refine our understanding and guide clinical decisions, especially considering patient history and risk factors.

